# Ode to my physician

**Published:** 2005

**Authors:** Shubhadarshini Singh

Too much to ask?
I sleep with your orders running in my blood
I wait to hear your words
In my veins can you hear the wariness?
Your orders coursing through my blood
Will they kill the grief and loneliness?
Will they pump up enough adrenaline to give me the will to fight and love my life?
In your face I see a touch of expectation: Of seeing me get better, thanks.

Do you know I sleep till noon in my bed at home?
Silent servants, distanced, happy to have a lazy lady
Serve me, breakfast, coffee with chemical sweetening
Give me the mail (What else but bills!)
Which had arrived while I slept, the worlds was up and about and alive it seems
How strange that no one can see the alarming changes in me the destruction has begun,
Progressive and painful and obvious only to me.

What are you waiting for:
A full-fledged MI? Help me, help me, for I need more than disturbed sleep cycles,
Glucometers and tests: What is the cause and what the effect:
Will you tell? Will you recognize my pain as disease not decadence
Didn't I tell you I read till 2 A.M. and toss till 4?
That I am becoming a creature of the night and as lone as a wolf?
Yet, you my doctor touch me with a steth and count my breath Or send me off to the lab to get AIDS probably.

Your idiosyncrasy of labelling all my ills as idiopathies!
Your CAT scans, ultrasound and EKGs, look so much like my daughter's geography
Charting my body. Don't these tests ever, ever tell you anything?
No, after all you're the master…of the slave of your wonderful laboratory
And your biomedical genii and the nuclear isotope
Are all only to give you the right to shrug off my unseen disease.

All I want is someone—someone like YOU should recognize my idiopathic despair. Or whatever. Today they have this wonderful theory, I read
That even mental disease can be treated biologically:
Dr Freud's psychoanalysis is out and the virus is in!
One day when I will snap, you won't be able to write me off So help me NOW! Balance all those humours going crazy inside me: dopamine, insulin, serotonin, lithium carbonate and Prozac may be? There must be something that will put me right
Or do they now always blame the virus for everything?

P.S. I am not a robot

